# Effects of the variation of metal substitution and electrolyte on the electrochemical reaction of metal hexacyanoferrates†

**DOI:** 10.1039/c8ra08091g

**Published:** 2018-11-06

**Authors:** Miyuki Asai, Akira Takahashi, Kazuki Tajima, Hisashi Tanaka, Manabu Ishizaki, Masato Kurihara, Tohru Kawamoto

**Affiliations:** Nanomaterials Research Institute, National Institute of Advanced Industrial Science and Technology (AIST) 1-1-1 Higashi Tsukuba 305-8565 Japan tohru.kawamoto@aist.go.jp; Department of Material and Biological Chemistry, Faculty of Science, Yamagata University 1-4-12 Kojirakawa-machi Yamagata 990-8560 Japan

## Abstract

Metal hexacyanoferrates (MHCFs), also called Prussian blue analogs, are known as electrochemical electrodes and are ion-adsorbent. To investigate the effect of the ionic radius of the adsorbate (cations adsorbed upon reduction) and the pore size of the adsorbent (porous electrode that stores cations upon reduction), we investigated the electrochemical reactions with various alkali cations and by changing the metal sites of the MHCFs. First, we succeeded in controlling the pore sizes of the MHCFs, where the lattice constant *a* could be estimated as *a* = 0.98*D*_sum_ + 7.21, where *D*_sum_ represented the sum of the ionic diameters of the metal M and Fe. Concerning the electrochemical reaction, the redox potential increased when the hydration energy of the adsorbate decreased, implying that the hydration energy of the adsorbate affected the stability of the reduced state. With cadmium hexacyanoferrate, which has a large pore size, the variation of the redox potential was suppressed in comparison to that with copper hexacyanoferrate, which has a small pore size. With Fourier transform-infrared (FT-IR) analysis before and after the redox reactions, Na^+^ insertion accompanied by H_2_O was presumed in the reduced state.

## Abstract 2

The redox potential of metal hexacyanoferrates (MHCFs), also called Prussian blue analogs, is qualitatively understood with the hydration energy of the cations in the supporting electrolyte.

## Graphical abstract

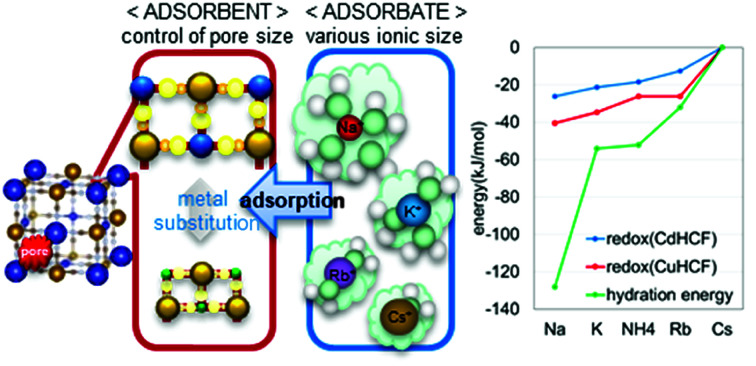

## 1 Introduction

Metal hexacyanoferrates (MHCFs), which are included in Prussian blue type-compounds, have been investigated concerning their physical and chemical properties over many years. In particular, because MHCFs have a porous network in their crystalline structure, their adsorption properties for small cations and molecules have been attracting attention.^1–4^ For example, because their selectivity for cesium cations is especially high, MHCFs can adsorb cesium, even in seawater.^5–10^ The electrochemical properties of MHCFs accompanied by adsorption and desorption of cations have also been investigated. The chemical formula for MHCFs is shown as A_*y*_M[Fe(CN)_6_]_*x*_, where A represents a monovalent cation such as Na, K, NH_4_, Rb, and Cs, and M indicates metal elements, *e.g.*, Fe, Cu, Mn, Zn, Co, Ni, and Cd. The valance number of M and Fe in MHCFs can be easily changed electrochemically if they are redox-active. Thus, MHCFs have been investigated for various electrochemical applications such as electrochromic devices (ECDs),^11–17^ secondary batteries,^18–25^ and the electrochemical recovery of cesium.^26,27^

It is important to systematically understand the dependence of these electrochemical properties on the kinds of MHCFs and on the kinds of adsorbate cations. For example, the substitution of M enables the control of color changes in ECDs, *e.g.*, Prussian blue, PB, changes its color from colorless to blue, green, and yellow.^28^ In contrast, nickel hexacyanoferrate (NiHCF) and copper hexacyanoferrate (CuHCF) change from yellow to colorless and from red to yellow, respectively.^28–30^ However, the dependences of redox potentials on the cations in the electrolyte have been reported.^26^ In the case of a secondary battery, the redox potential greatly affects the electromotive force.

The redox reactions of MHCFs are strongly connected to the adsorption and desorption of the monovalent cations. The electrochemical reaction with the redox of Fe cations is described as

1 M^II^[Fe^III^(CN)_6_]_*x*_ + *y*A^+^ + *y*e^−^ ↔ A_*y*_M^II^[Fe^II^(CN)_6_]_*x*_.

In this reaction, A^+^ is adsorbed at an interstitial pore confined by a cubic structure consisting of eight metals and twelve CN groups.^31^ In other words, the reduced and oxidized reactions are accompanied by A^+^ adsorption and A^+^ desorption, respectively. Therefore, the redox potential is likely to have a close relation with both the hydration energy and the adsorption energy into the MHCFs.

**Fig. 1:**
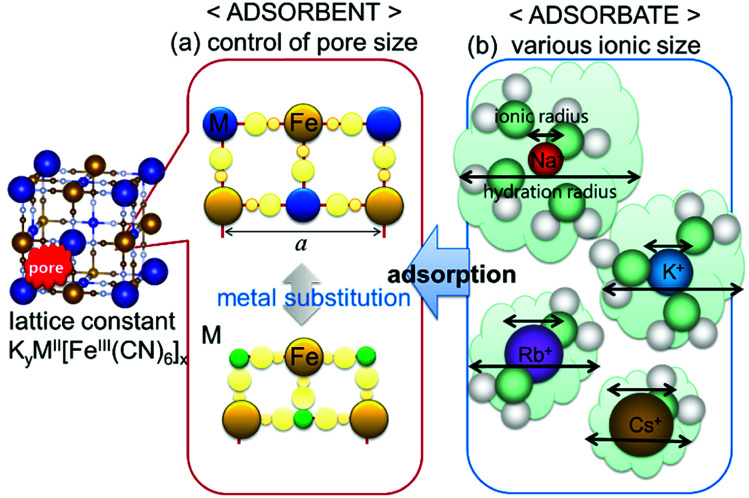
Schematic view of parameters that possibly affect adsorption behavior: (a) pore size of MHCF controlled by metal replacement and (b) various ion sizes as adsorbate.

Considering the effect of the adsorption energy on the electrochemical reaction, we focused on the lattice constant of MHCF crystalline structure because the lattice constant is usually strongly related to the pore size in the crystalline structure, which would affect the electrochemical adsorption and desorption properties. The crystalline structure of MHCFs is shown in Fig. 1. With a simple assumption, the lattice constant, *a*, and the pore size, *d*_pore_, may be expected from the ionic radii and the molecular size of the cyano group as

2 *a*_exp_ = 2(*r*_M_ + *r*_Fe_) + *α*,

3 = *D*_sum_ + *α*,

and4
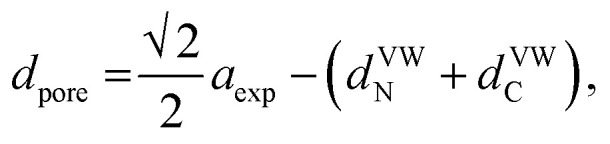
where *a*_exp_, *r*_M_, and *α* represent the expected lattice constant, the ionic radius of the M ion, and the constant term corresponding to the size of the CN group, respectively. The sum of the diameters of M and Fe, *D*_sum_, is calculated as 2(*r*_M_ + *r*_Fe_). *d*^VW^_N_ and *d*^VW^_C_ represent the van der Waals radii for the N and C ions, respectively. In general, in terms of the ion adsorption behavior of porous materials, the size compatibility of the pore size of the adsorbent with the ionic radius of the adsorbate is quite important. However, in the case of MHCFs, the hydration radius of the adsorbate is often needed to understand the adsorption behavior.^26^ This implies a possibility that the adsorption behavior of MHCFs is governed by another factor.

In this study, we investigated the effects of the lattice constants and the ionic radii of the adsorbate cations on the electrochemical adsorption behavior of MHCFs. In the previous studies by other researchers, the effect on the adsorbate cation dependence was mainly focused.^32–34^ We investigated more extensively with the variation of the lattice constant.

First, we synthesized various MHCFs with differing metal elements to confirm the dependence of the lattice constant on the metal elements. Next, the electrochemical measurements of cadmium hexacyanoferrate (CdHCF) and copper hexacyanoferrate (CuHCF) in various supporting electrolytes, namely, various adsorbate cations, were investigated. The reason for our choice of CdHCF and CuHCF is that the lattice constant of CdHCF was the largest and that of CuHCF was the smallest among our synthesized MHCFs. During the investigation, the hydration energy of the adsorbate in the electrolyte before adsorption seemed to be important. Finally, we discussed the configuration at the adsorption using the infrared (IR) spectra of the samples before and after the electrochemical reaction.

## 2 Methods

### 2.1 Synthesis of MHCFs

We focused on MHCFs without monovalent cation A at the fresh condition before the electrochemical reaction in order to exclude the effect of the initially implemented cations. The chemical formula of the MHCFs is expected to be M^II^[Fe^III^(CN)_6_]_0.66_. To confirm the effect of the metal substitution, Cd, Fe, Mn, Co, Zn, Cu, and Ni were used for the M^II^ site. All samples were synthesized in the same way: MCl_2_ in H_2_O (0.46 mol mL^−1^, 5 mL) and K_3_[Fe(CN)_6_] in H_2_O (0.30 mol L^−1^, 5 mL) were mixed at room temperature and shaken with 1000 rpm for 24 h at room temperature using a shaking incubator (SI-300C, AS ONE). The powder was obtained through aqueous rinsing by centrifugation three times, followed by drying at room temperature in vacuum.

### 2.2 Characterization of MHCF

The chemical composition of the obtained MHCFs was experimentally evaluated as follows: the composition ratio of K, Fe, and M (M = Cd, Fe, Mn, Co, Zn, Cu, and Ni) was evaluated as follows: the pre-decomposition of the samples by a microwave digestion system (Multiwave 3000, Perkin Elmer Inc.), followed by the investigation of the concentration of each element by a microwave plasma-atomic emission spectrometer (4100MP-AES, Agilent). The water content was obtained by using a thermogravimeter (Thermo Plus EVO, Rigaku Corp.). The concentrations of C and N in FeHCF were measured with an elemental analyzer (2400 II, PerkinElmer) after decomposition to determine the CN content. The chemical compositions of other materials were estimated by assuming that the ratio of iron and cyanide was six, *i.e.*, the CN group was assumed to be included only as the form of [Fe(CN)_6_].

The crystalline structures of MHCFs were identified by X-ray diffraction analysis with D2 phaser Bruker AXS Inc. The lattice constant was obtained by the Pawley method. For the calibration of angles, the silicon powder NIST 640e was mixed for an internal standard. The crystallite size was calculated as5
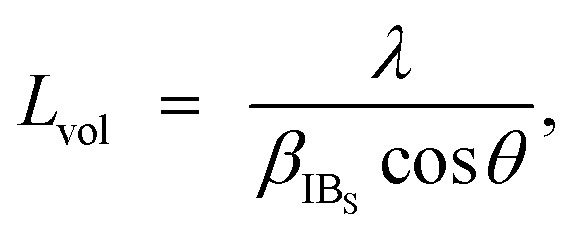
where *L*_vol_, *β*_IB_S__, *θ*, and *λ* represent the crystalline structure size, half-value integration, diffraction angle, and wave length, respectively.^35^ Half-value integration is a value where the peak area is divided by the integration of the peak top.

The specific surface area was evaluated by Brunauer–Emmett–Teller (BET) analysis using an automatic specific surface area evaluation system (BELSORP-max, Microtrac BEL Inc.). Sample images were taken using a field emission scanning electron microscope (FE-SEM, S-4800; Hitachi High Technologies Corp.).

### 2.3 Ink preparation

For the preparation of the thin film electrode for the electrochemical analysis, we prepared an ink of CdHCF and CuHCF. CdHCF (250 mg) and an aqueous solution of 5 wt% polyvinyl alcohol (250 mg) were put into H_2_O (4.5 g) and stirred overnight at room temperature using a magnetic stirrer. CuHCF was prepared in the same way. We prepared two kinds of electrodes with different thicknesses. As the substrate for the electrode, ITO (2.5 cm × 2.5 cm) was treated with plasma (HARRICK PLAZMA/RDEC Corp.). The thinner electrodes were prepared by coating with the MHCF ink (M = Cd, Cu, 300 μL) using a spin coater (ACT300A, ACTIVE). The rotation speed and the time was set to 500 rpm for 10 s, respectively, followed by 1000 rpm for 10 s, respectively. The thicker electrodes were prepared for IR spectrum measurement. Ink (50 μL) was placed on ITO (10 cm × 5 cm) and coated using an applicator (Hohsen Corp.) with five round trips.

### 2.4 Cyclic voltammetry

The thinner electrodes were evaluated by cyclic voltammetry analysis (ALS6115D, BAS). The reference electrode was Ag/AgCl with a saturated KCl aqueous solution and the counter electrode was a platinum wire. Cyclic voltammetry measurements were performed at 5 mV s^−1^ in an AX aqueous solution (A = Na^+^, K^+^, NH_4_^+^, Rb^+^, and Cs^+^, X = SO_4_^2−^, Cl^−^, and NO_3_^−^, 10 mM L^−1^).

### 2.5 IR spectra before and after the electrochemical treatment

To clarify the reaction in the electrochemical treatments, we evaluated the IR spectra before and after treatment. In order to obtain strong IR absorbance, we used the thicker electrodes. The thicker electrodes were reduced at 0 V *vs.* Ag/AgCl for 30 s in an AX aqueous solution (A = Na^+^, K^+^, NH_4_^+^, Rb^+^, and Cs^+^, and X = SO_4_^2−^, Cl^−^, NO_3_^−^, 10 mM L^−1^). After N_2_ drying, Fourier transform-infrared (FT-IR) spectra were investigated using NICOLET iS5 (Thermo SCIENTIFIC).

## 3 Results & discussion

### 3.1 Relation between the lattice constant and type of metal

**Fig. 2:**
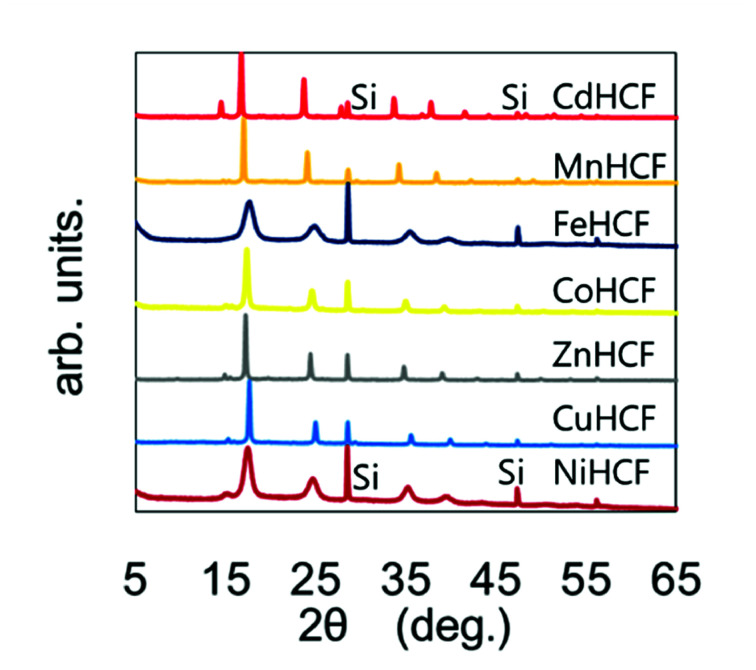
XRD patterns of various MHCFs. The peaks with the notation “Si” represent those from silicon for calibration of the peak position.

**Fig. 3:**
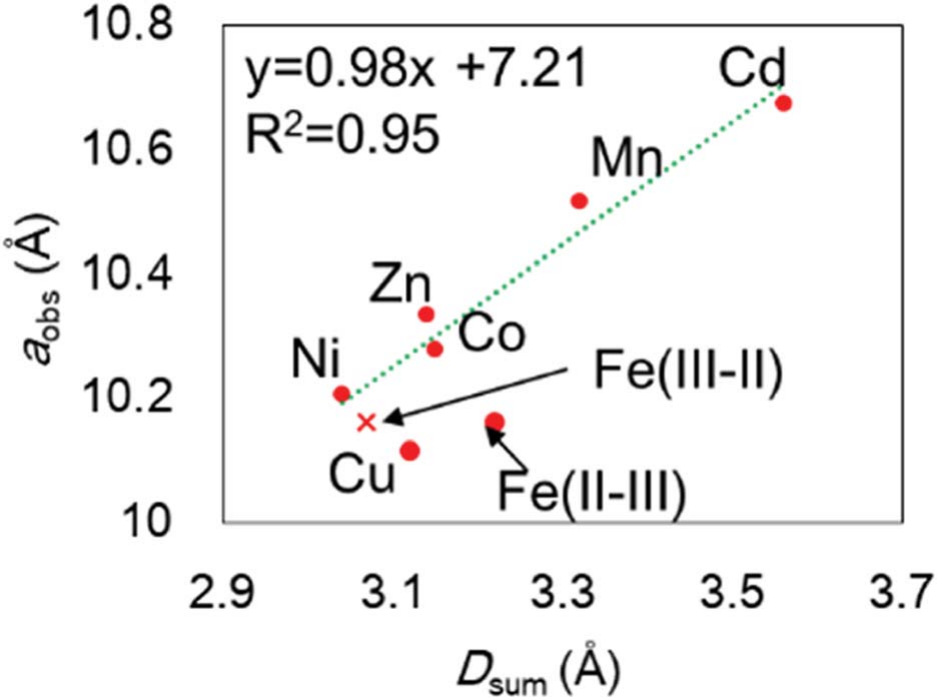
Relation between the experimentally observed lattice constant, *a*_obs_, and the sum of the ionic diameters of the metal elements, *D*_sum_. The element notation M shows that in M[Fe(CN)_6_]_*x*_. Fe(iii–ii) and Fe(ii–iii) represent Fe(iii)[Fe(ii)(CN)_6_]_*x*_ and Fe(ii)[Fe(iii)(CN)_6_]_*x*_, respectively. The broken line is drawn with fitting except for CuHCF and with Fe(iii–ii) instead of Fe(ii–iii).

The chemical compositions of the MHCFs are shown in Table 1. All MHCFs had similar chemical compositions, having little potassium content as expected. The X-ray diffraction analysis (XRD) patterns are shown in Fig. 2, indicating that all the MHCFs could be analyzed as cubic structures, *Fm*3̄*m*. The experimentally obtained lattice constants, *a*_obs_, were almost proportional to the sum of the diameters of the metal ions, *D*_sum_, as shown in Fig. 3, except for CuHCF and FeHCF. Concerning FeHCF, the Fe^II^HCF^III^ initially synthesized, Turnbull's blue, would be transformed into Fe^III^HCF^II^, Prussian blue, through the electron transfer between Fe ions. If the charge transfer between Fe's, FeHCF also satisfies the proportional relation. With the exception of CuHCF, the relation between *a*_obs_ and *D*_sum_ was well fitted the proportional function, *a*_obs_ = 0.98*D*_sum_ + 7.21. Because the slope was nearly 1, *a*_obs_ was found to be elongated as long as *D*_sum_ increased.

**Table 1:** Information on the synthesized MHCFs, A_*y*_M[Fe(CN)_6_]_*x*_·*z*H_2_O, where *r*_M_, *r*_sum_, and *a*_obs_ represent the ionic radius of M, the sum of the diameters of M and Fe (2*r*_M_ + 2*r*_Fe_), and the observed lattice constant, respectively. The bottom row represents the case for Prussian blue for reference

M		*r*_M_ (Å)	Fe		*r*_Fe_ (Å)	Chemical composition	*D*_sum_	*a*_obs_ (Å)
Cd	2+	1.09	Fe	3+	0.69	K_0.12_Cd[Fe(CN)_6_]_0.69_·3.6H_2_O	1.78	10.67
Mn	2+hs	0.97				K_0.07_Mn[Fe(CN)_6_]_0.69_·3.5H_2_O	1.66	10.52
Fe	2+hs	0.92				K_0.08_Fe[Fe(CN)_6_]_0.58_·2.5H_2_O	1.61	10.16
Co	2+hs	0.89				K_0.06_Co[Fe(CN)_6_]_0.65_·3.9H_2_O	1.58	10.28
Zn	2+	0.88				K_0.07_Zn[Fe(CN)_6_]_0.58_·3.2H_2_O	1.57	10.33
Cu	2+	0.87				K_0.04_Cu[Fe(CN)_6_]_0.68_·3.1H_2_O	1.56	10.12
Ni	2+	0.83				K_0.15_Ni[Fe(CN)_6_]_0.63_·4.2H_2_O	1.52	10.21
Fe	3+hs	0.79	Fe	2+ls	0.75		1.54	10.16

In the case of CuHCF, the reason of the shrinkage of the lattice constant is not unclearer, but it would originate from the pseudo five-coordinated or four-coordinated environment of Cu^2+^ cations. We synthesized material with a formula of K_0.04_Cu[Fe(CN)_6_]_0.66_. With such a formula having [Fe(CN)_6_] vacancies with its high density, Cu^2+^ cations are surrounded by various numbers of CN groups.^36^ Therefore, the number of coordinating surrounding ligands around Cu^2+^ depends on the number of coordinated hydrated molecules.

The lattice constant could be controlled by the ionic radii of the metal elements. The difference between CdHCF and CuHCF, which have respectively the largest and smallest lattice constant, is 0.55 Å. The expected pore sizes from eqn (4) are 4.29 Å for CdHCF and 3.91 Å for CuHCF. The difference implies that 32% of pore volume expansion is expected. Note the correlation between the BET surface area and the lattice constant, as shown Fig. S1 and Table S1 in the ESI.†

**Fig. 4:**
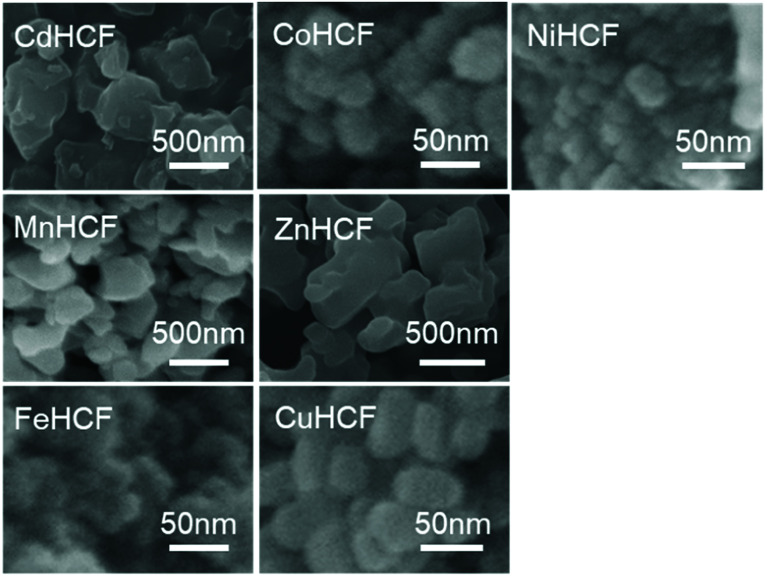
SEM images of MHCFs.

**Fig. 5:**
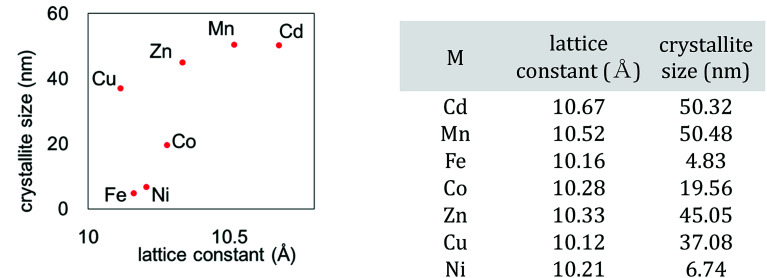
Relation between crystallite size and lattice constant.

We also investigated the particle size of the obtained MHCFs. Fig. 4 shows the SEM images. All samples were obtained as small particles less than 1 μm. However, in detailed consideration, we found that the crystallite size obtained from Scherrer analysis with the XRD patterns was dependent on the lattice constant, as shown in Fig. 5. It was found that the particle size increased as the lattice constant increased. The reason for the relation is unclear.

### 3.2 Electrochemical properties of CdHCF and CuHCF

Next, we investigated the electrochemical properties of CdHCF and CuHCF. As described above, CdHCF had the largest lattice constant and pore size, whereas CuHCF had the smallest among the obtained MHCFs.

**Fig. 6:**
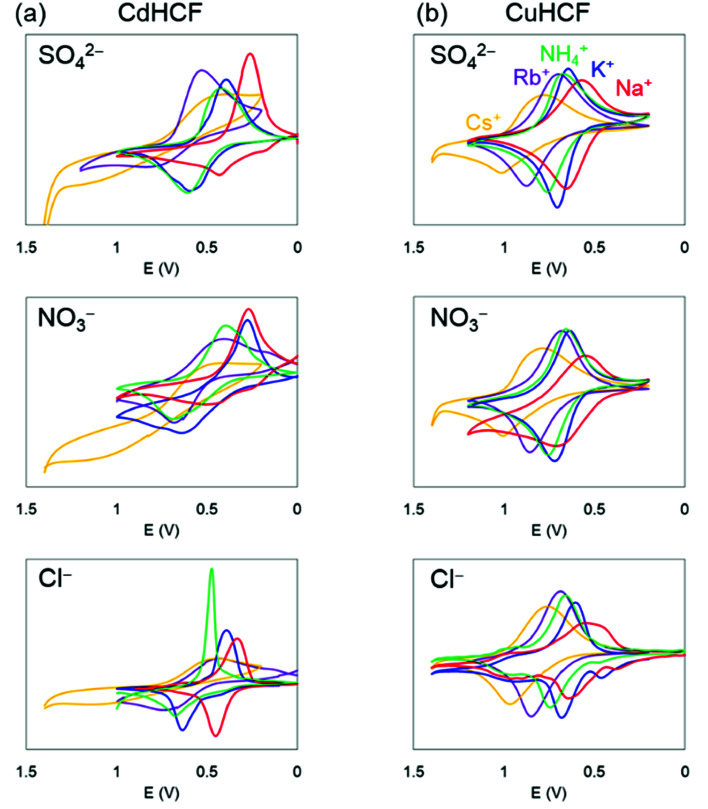
Cyclic voltammetry at 5 mV s^−1^ in aqueous solution with supporting electrolyte AX (A = K^+^, Na^+^, NH_4_^+^, Rb^+^, and Cs^+^, X = SO_4_^2−^, Cl^−^, and NO_3_^−^, 10 mM L^−1^): (a) CdHCF and (b) CuHCF. The vertical axis indicates the renormalized value by the injected charge during the process of the potential increase, to avoid the difference of the film thickness among the samples.

The cyclic voltammograms are shown in Fig. 6. These trends were almost the same. Namely, there was one main redox peak at each potential sweep for oxidation and for reduction. The redox potential for the main peaks obtained from the voltammograms are shown in Table 2, where the redox potentials were calculated as the average of the largest oxidized and reduced peaks. For comparison, the ionic radii and hydrated radii of Na^+^, K^+^, NH_4_^+^, Rb^+^, and Cs^+^ are also shown in Table 2. The redox potential depends on the cation species, where the order is Na^+^, K^+^, NH_4_^+^, Rb^+^, and Cs^+^ in ascending order, independent of both the species of M^2+^ in the MHCFs and the anion in the electrolyte. This result indicates that the reduced state with adsorption of cations into the lattice becomes more stable than the oxidized state when the hydration radius decreases. Since lithium has higher hydration energy than the other cations, −519 kJ mol^−1^,^37^ its redox potential would be lower than those.

**Table 2:** The ionic radii, hydrated radii, and hydrated energies of Na^+^, K^+^, NH_4_^+^, Rb^+^, and Cs^+^ in the literature and the redox potentials obtained from the cyclic voltammograms. The ion radius, the hydration radii, and the hydration energies were quoted from a previous report^37,41^

			Na^+^	K^+^	NH_4_^+^	Rb^+^	Cs^+^	Average	
Ion radius (Å)			0.95	1.33	1.48	1.48	1.69		
Hydration radius (Å)			3.58	3.31	3.31	3.29	3.29		
Hydration energy (kJ mol^−1^)			−419	−345	−343	−323	−291		
Redox potential (V)	CdHCF	SO_4_^2−^	0.39	0.52	0.58	0.59	0.80	0.57	0.57
		Cl^−^	0.39	0.45	0.54	0.54	0.81	0.55	
		NO_3_^−^	0.35	0.50	0.53	0.69	0.80	0.57	
		Avg.	0.38	0.49	0.55	0.61	0.80		
	CuHCF	SO_4_^2−^	0.60	0.64	0.77	0.77	0.86	0.73	0.73
		Cl^−^	0.63	0.68	0.71	0.77	0.90	0.74	
		NO_3_^−^	0.61	0.68	0.72	0.79	0.90	0.74	
		Avg.	0.61	0.67	0.73	0.78	0.89		
	Difference between CdHCF and CuHCF	SO_4_^2−^	0.21	0.13	0.20	0.18	0.07		
		Cl^−^	0.24	0.23	0.17	0.23	0.09		
		NO_3_^−^	0.26	0.18	0.18	0.10	0.10		

**Fig. 7:**
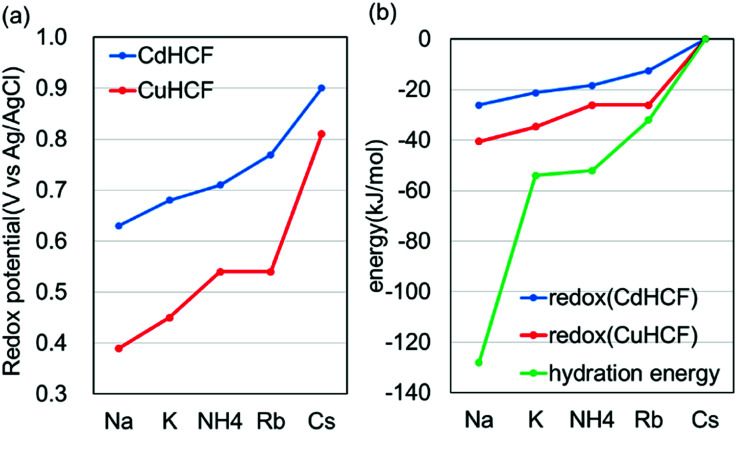
Dependence of the redox potential on the type of adsorbent and the adsorbate cations. (a) The relation between the adsorbates and the redox potential and (b) adsorbate-dependence of redox potential, converted into energy (kJ mol^−1^), and that of the hydration energy, where the energies for Cs are set as the reference value.

For more quantitative analysis, the dependence of the redox potential on the variation of the adsorbents (=MHCFs) and the adsorbate (=monovalent cations) is shown in Fig. 7. Fig. 7(a) shows that the redox potential increased as the ionic radius of the adsorbate increased, and thus the slope of the redox potential increases in the range of large ionic radii, meaning that the electrochemical recovery of Cs is possible.^26^

Changing the lattice constant of the adsorbent did not show qualitative differences. But in the case of the CuHCF, the variation of the redox potential with change in the adsorbate was larger than in the case of CdHCF, implying the CuHCF is more suitable for Cs adsorption. The small variation of the redox potential with CdHCF would be caused by the large pore size. Because MHCFs are crystalline structures consisted of ions, the main contribution on the adsorption energy would be the Coulomb energy between MHCFs and the adsorbate cations. Therefore, the effect on the kinds of cation would be suppressed with the larger pore size.

We didn't evaluate mixed-metals hexacyanoferrates, including a couple of metal cations at the site M. But it wouldn't be caused an issue. The mixed metal hexacyanoferrate would have the lattice constant of among the two-original metal hexacyanoferrates. For example, (Fe,Cd)HCF have a lattice constant between FeHCF and CdHCF depending on its metal ratio.^38^ The mixed metal hexacyanoferrate would show a property correlated with its lattice constants.

Fig. 7(b) shows the dependence of the redox potential and that of the hydration energy on the variation of the adsorbate monovalent cations. To do a quantitative comparison, the redox potential was converted into units of kJ mol^−1^. It was found that the variation of the redox potential was smaller than that of the hydration energy. It seems, therefore, that the effect of the hydration energy on the redox potential is softened by some effects. In addition, it would be reasonable to conclude that the effect of the adsorption energy of the adsorbates into the adsorbent is smaller than the difference of the hydration energy. If the adsorption energy is more effective than the hydration energy, dependence of the redox potential on the variation of the adsorbates would be larger than the difference of the hydration energy.

Concerning with other solvents, we think the tendency is not so different from the aqueous solution, because the order of the solvation energy of monovalent cations are the same, *e.g.* for dimethyl sulfoxide,^39^ and methanol,^40^ although quantitative difference exists.

### 3.3 FT-IR spectra before and after the redox reactions

**Fig. 8:**
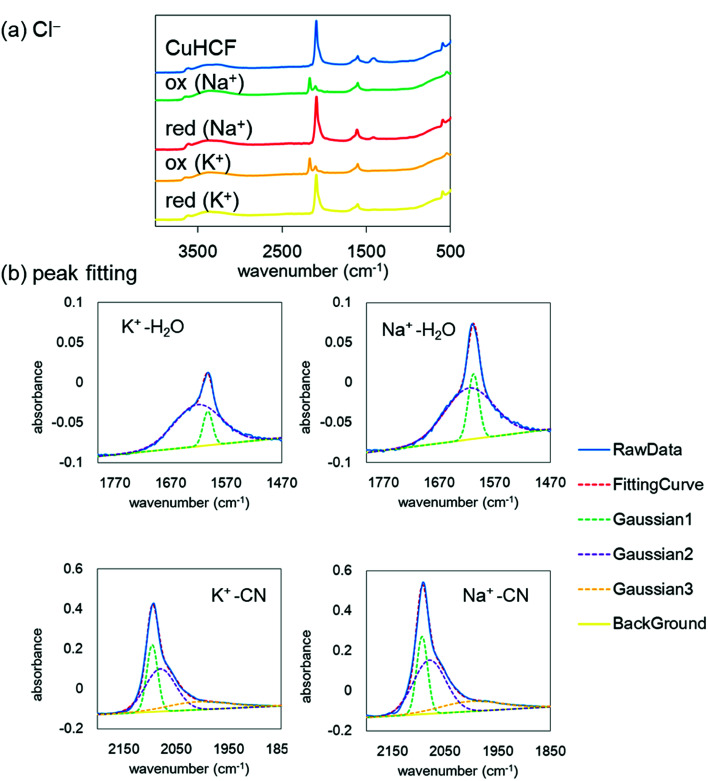
(a) IR spectra of CuHCF in ACl before and after reduction. (b) Enlarged view of the reduced state at ∼1600 cm^−1^ and 2100 cm^−1^, corresponding to the bending mode of H_2_O and the stretching mode of CN^−^, respectively. K and Na indicate the alkali cations in the supporting electrolyte. The solid line represents a raw data. The broken lines show fitting curves with Gaussians and background straight line. The fitting was done by the least-square method. The blue solid line and the red broken line were completely overlapping.

Finally, to understand the difference of the adsorption configuration with the variation of adsorbate cations, we evaluated the IR spectra before and after the redox reactions with NaCl or KCl in the supporting electrolyte. Fig. 8(a) shows the FT-IR spectra of the CuHCF film at the initial state, the oxidized state, and the reduced state in the electrolyte including Na^+^ or K^+^. The tendency of the spectra is almost the same. However, the CN stretching peak was shifted from 2193 cm^−1^ at the oxidized state to 2092 cm^−1^ at the reduced state, indicating the reduction of [Fe(CN)_6_]^3−^ to [Fe(CN)_6_]^2−^.

The spectrum in the oxidized form shows clearly two peaks. The low energy peak, around 2090 cm^−1^, accounts for reduced species Fe(ii)–CN–Cu, indicating the incomplete oxidization.

Here, we focus on the peaks at about 1600 cm^−1^ and 2100 cm^−1^ after the reduction with the adsorption of Na^+^ or K^+^. The enlarged views are shown in Fig. 8(b). The peak at around 1600 cm^−1^ corresponds to the H_2_O bending mode and that at around 2100 cm^−1^ corresponds to the CN stretching mode. Both peaks are well reproduced with a few Gaussians. The Gaussians were represented for broken lines in Fig. 8(b). In general, a narrow component of the H_2_O peak indicates an unchanging environment around H_2_O, such as the strongly bound H_2_O in the crystalline structure, and the broad peak corresponds to the variation of the environment, such as in liquid water, with the hydrogen bond network changing from moment to moment. With this consideration, we evaluated the amount of strongly bound H_2_O in the CuHCF crystalline structure by comparing the peak ratios of the H_2_O and CN peaks. Table 3 shows the peak area ratio of the H_2_O peak and that of the CN peak. The results indicate that the reduced state with the Na^+^ contained more strongly bound H_2_O than that with K^+^. In the Na^+^ case, we speculate that the H_2_O molecules were bound in the interstitial sites accompanying Na^+^, as well as in the open-metal sites at the [Fe(CN)_6_] vacancies. However, in the K^+^ case, it seems that H_2_O molecules are only bound in the open-metal sites, consistent with a previous report.^42^ The difference between Na^+^ and K^+^ would thus originate from the ionic radius difference.

**Table 3:** The peak area ratio of the narrow component at about 1600 cm^−1^, corresponding to the H_2_O bending mode, to that at 2100 cm^−1^, corresponding to the CN stretching mode

	Cl^−^	NO_3_^−^	SO_4_^2−^	Average
K	0.038	0.033	0.036	
Na	0.064	0.077	0.048	
Na/K	1.7	2.3	1.3	1.8

This difference would be one of the reasons for the drastic suppression of the energy for redox reactions in the case of Na^+^. As shown in Fig. 7(b), the difference between the energy for redox reaction and the hydration energy is greatest in the case of Na^+^. Na^+^ cations would be adsorbed into the crystalline structure accompanied by H_2_O. In this case, the loss of the hydration energy at the adsorption may be decreased, resulting in the large difference between the hydration energy and the energy for redox reactions.

Finally, we should note that the vacancies of [Fe(CN)_6_]^*α*−^. Because the concentration of the [Fe(CN)_6_] sites was set to ∼1/3 for all of MHCFs, the effect of the vacancies are not discussed. The effect of the vacancies was described in another article, where we revealed the vacancies are essential as the percolation path for the cation to penetrate into the MHCF particles.^42^

## 4 Conclusion

The redox potential of MHCFs strongly depends on the kinds of monovalent cations in the supporting electrolyte. The difference in the hydration energy is consistent with the order of the redox potential with change in the monovalent cation adsorbate in the supporting electrolyte. In the case of Na^+^, it seems that H_2_O molecules remain in the interstitial pores with Na^+^. In this paper, we also evaluated the effect of the lattice expansion. It is found that the lattice expansion does not cause the quantitative change for the order of the adsorption energy with monovalent cations, but affect on the energy quantitatively. In the combination with the consideration of [Fe(CN)_6_] vacancies described in another articles,^42^ the adsorption energy of monovalent cations in MHCFs are well understood. It also help the researches for the design of the secondary batteries with MHCF electrodes.

## Author contributions

All authors have given approval to the final version of the manuscript.

## Conflicts of interest

There are no conflicts to declare.

## Abbreviations

MHCF: Metal hexacyanoferrate
CuHCF: Copper hexacyanoferrate
CdHCF: Cadmium hexacyanoferrate

## Supplementary Material

RA-008-C8RA08091G-s001
