# Supplementary material for: Effects of the variation of metal substitution and electrolyte on the electrochemical reaction of metal hexacyanoferrates
Source: RSC Adv. 2018 Nov 6;8(65):37356–64. doi: 10.1039/c8ra08091g (PMC9089244; doi:10.1039/c8ra08091g)
Supplement: RA-008-C8RA08091G-s001 [file RA-008-C8RA08091G-s001.pdf]

Electronic Supplementary Material (ESI) for RSC Advances.
This journal is © The Royal Society of Chemistry 2018

Supporting Information for

Effects of the variation of metal substitution and that of ad-sorbate on electrochemical reaction of metal hexacyanoferrates

Miyuki Asai[a,b], Akira Takahashi[a], Kazuki Tajima[a], Hisashi Tanaka[a], Manabu Ishizaki[b], Masato Kurihara[a,b], and Tohru Kawamoto[a,b] *

a. Nanomaterials Research Institute, National Institute of Advanced Industrial Science and Technology (AIST), 1-1-1 Higashi, Tsukuba, Ibaraki 305-8565, Japan. E-mail; tohru.kawamoto@aist.go.jp

b. Department of Material and Biological Chemistry, Faculty of Science, Yamagata University, 1-4-12 Kojirakawa-machi, Yamagata 990-8560, Japan,

1. Specific surface area analysis

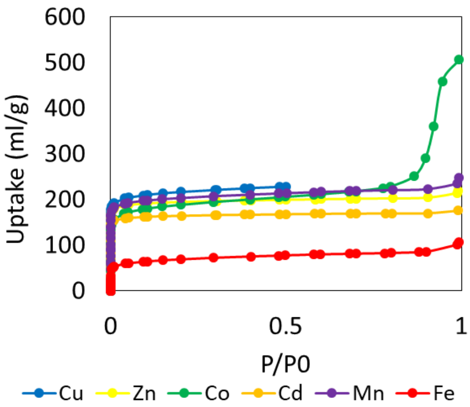

Figure SI 1. N_2 adsorption of MHCF

Table SI1. Amount of monomolecular layer adsorption and specific surface area.

	Vm (cm^3/g)	S_{BET} (cm^2/g)
Cd	148	645
Fe	57	248
Mn	181	786
Co	162	705
Zn	168	731
Cu	196	852
Ni	-	-

2. CuHCF and CdHCF films coated using spin-coating and applicator.

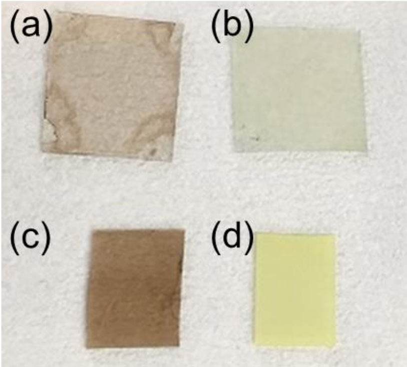

Figure SI 2. MHCF thin films for cyclic voltammetry and IR measurement (a) CuHCF by spin-coating (b)CdHCF by applicator (c) CuHCF by spin-coating (d)CdHCF by applicator

3. Parameters of Gaussian function for IR spectra fitting

Gaussian function; $y=A\exp(-(x-x_0)^2/2\alpha^2)$

Area of peak; $S=\sqrt{2\pi}A\alpha$

Table SI2. Parameters of Gaussian function for IR spectra.

			A	α	x_0	S			a	b
CN	CN-1	Na	0.426	9.85	2095	10.5	linear	Na	-1.7E-04	0.245
		K	0.458	9.60	2096	11.0		K	-1.5E-04	0.198
	CN-2	Na	0.243	28.9	2081	17.6				
		K	0.262	29.8	2081	19.6				
	CN-3	Na	0.0579	56.0	2012	8.12				
		K	0.0681	58.1	2005	9.92				
H_2O	H_2O-1	Na	0.0565	9.64	1606	1.36	linear	Na	-9.1E-05	0.0830
		K	0.0519	8.56	1603	1.11		K	-8.5E-05	0.0673
	H_2O-2	Na	0.0597	46.1	1617	6.90				
		K	0.0599	46.5	1617	6.97				
